# Estimation of differential renal function on routine abdominal imaging employing compressed-sensed contrast-enhanced MR: a feasibility study referenced against dynamic renal scintigraphy in patients with deteriorating renal retention parameters

**DOI:** 10.1007/s00261-023-03823-2

**Published:** 2023-02-02

**Authors:** Victor Schulze-Zachau, David J. Winkel, Felix Kaul, Theo Demerath, Silke Potthast, Tobias J. Heye, Daniel T. Boll

**Affiliations:** 1 Department of Radiology, University Hospital of Basel, 4031 Basel, Switzerland; 2 Department of Nuclear Medicine, University Hospital of Basel, Basel, Switzerland; 3 Neuroradiology Clinic, University Medical Center Freiburg, Freiburg, Germany; 4 Department of Radiology, Spital Limmattal, Schlieren, Switzerland

**Keywords:** Contrast media, Renal function, Image processing, Golden-angle radial sparse parallel (GRASP)

## Abstract

**Purpose:**

To assess whether high temporal/spatial resolution GRASP MRI acquired during routine clinical imaging can identify several degrees of renal function impairment referenced against renal dynamic scintigraphy.

**Methods:**

This retrospective study consists of method development and method verification parts. During method development, patients subject to renal imaging using gadoterate meglumine and GRASP post-contrast MRI technique (TR/TE 3.3/1.6 ms; FoV320 × 320 mm; FA12°; Voxel1.1 × 1.1x2.5 mm) were matched into four equally-sized renal function groups (no-mild-moderate-severe impairment) according to their laboratory-determined estimated glomerular filtration rates (eGFR); 60|120 patients|kidneys were included. Regions-of-interest (ROIs) were placed on cortices, medullary pyramids and collecting systems of bilateral kidneys. Cortical perfusion, tubular concentration and collecting system excretion were determined as Time_Cortex=Pyramid_(sec), Slope_Tubuli_ (sec^−1^), and Time_Collecting System_ (sec), respectively, and were measured by a combination of extraction of time intensity curves and respective quantitative parameters. For method verification, patients subject to GRASP MRI and renal dynamic scintigraphy (99mTc-MAG3, 100 MBq/patient) were matched into three renal function groups (no-mild/moderate-severe impairment). Split renal function parameters post 1.5–2.5 min as well as MAG3 TER were correlated with time intensity parameters retrieved using GRASP technique; 15|30 patients|kidneys were included.

**Results:**

Method development showed differing values for Time_Cortex=Pyramid_(71|75|93|122 s), Slope_Tubuli_(2.6|2.1|1.3|0.5 s^−1^) and Time_Collecting System_(90|111|129|139 s) for the four renal function groups with partial significant tendencies (several *p*-values <  0.001). In method verification, 29/30 kidneys (96.7%) were assigned to the correct renal function group.

**Conclusion:**

High temporal and spatial resolution GRASP MR imaging allows to identify several degrees of renal function impairment using routine clinical imaging with a high degree of accuracy.

## Introduction

Nephrectomy is a frequently performed medical procedure, either in an oncologic context or in living donor renal transplantation scenarios^1^, ^2^. The surgical risk of nephrectomy is low, however, living with a single kidney has lifelong implications. The resultant single-kidney condition may impact overall life expectancy and quality of life, incur future healthcare costs and is associated with the risk of developing hypertension and end-stage kidney disease^2^, ^3^. Detecting any type of pre-existing renal impairment or identifying renal function parameters predicting future dysfunction of the remaining contralateral kidney may be considered important information prior to nephrectomy and may impact choice and timing of the surgical procedure offered to the patient and potential follow-up care.

Glomerular filtration rate (GFR) is a widely accepted measure of renal function. GFR can be estimated (eGFR) using laboratory tests, such as serum creatinine levels. The testing is cheap and widely available, but it is not sensitive in early stages of nephron damage and associated with high measurement variability. Furthermore, no single-kidney information can be obtained by means of serum laboratory testing.

Conventional dynamic renal scintigraphy with additional estimation of MAG3 TER (mercaptoacetyltriglycine tubular extraction rate) is a well-established method and allows for individual kidney evaluation. However, it is characterized by low spatial resolution and necessarily exposes the patients to ionizing radiation. No other functional and only little morphologic information can be obtained via scintigraphy.

Lastly, MRI has been performed to evaluate renal function. The gadolinium-based contrast agent (GBCA) gadoterate meglumine is freely filtrated in the glomeruli and undergoes neither reabsorption nor active secretion in the renal tubuli – characteristics shared by widely accepted tracers of renal function such as inulin^4^. It therefore qualifies as a potential biomarker for the assessment of renal function. MRI renography offers the possibility to assess kidneys separately and even evaluate sub-regions of individual kidneys. Until now, however, functional assessment with MRI has mainly been performed as DCE MRI with dedicated protocols and an additional dosis of GBCA. Typically, compartmental models and subsequent kinetic modeling need to be applied to the MRI data, requiring conversion from relative non-calibrated MR signal intensities to GBCA concentrations. Non-linearity between MRI signal and GBCA concentration as well as the multiplicity of steps in data analyses increase complexity and constitute possible sources of error^5^.

The recently introduced golden-angle radial-sparse parallel (GRASP) MRI sequence combines a fat-saturated T1-weighted radial stack-of-stars 3D gradient-echo sequence with compressed sensing and parallel-imaging reconstruction. GRASP post-hoc reconstructions thereby simultaneously increase temporal as well as spatial resolution for DCE MRI without substantial deterioration of overall image quality under free-breathing conditions of already acquired GRASP raw data sets, beyond the capabilities of traditionally employed post-contrast imaging sequences^6^, ^7^.

Our study sought to exploit this simultaneous increase in temporal and spatial resolution to precisely quantify the passage of gadolinium-based contrast material as tracer through vascular, tubular and collecting system compartments in order to assess differential renal function while avoiding estimation of contrast agent concentration. Therefore, patient populations with several levels of renal function impairment, image acquisition under routine diagnostic circumstances and comparison to dynamic renal scintigraphy as well-established standard to evaluate single organ functionality were chosen.

This retrospective pilot study was designed to test the hypothesis that high temporal and spatial resolution GRASP MRI allows to reliably identify several degrees of differential renal function impairment using routine clinical imaging data.

## Methods

This retrospective study was approved of by the institutional review board of the University Hospital of Basel. Informed patient consent was provided by all study subjects.

### Study population

This study consists of a Method Development as well as a Method Verification part.

For Method Development, the local radiology information system (RIS) was retrospectively searched for abdominal MRI examinations with DCE-MRI employing GRASP and weight-adapted doses of administered gadoterate meglumine performed between 03/2016 and 01/2021. Patients where included into the initial search when the following inclusion criterion was met: availability of serum creatinine measurement at the day of the MRI examination; excluded from the initial search were patients with multicystic or neoplastic kidney disease as well as presence of fresh renal infarctions or inflammatory foci.

**Fig. 1:**
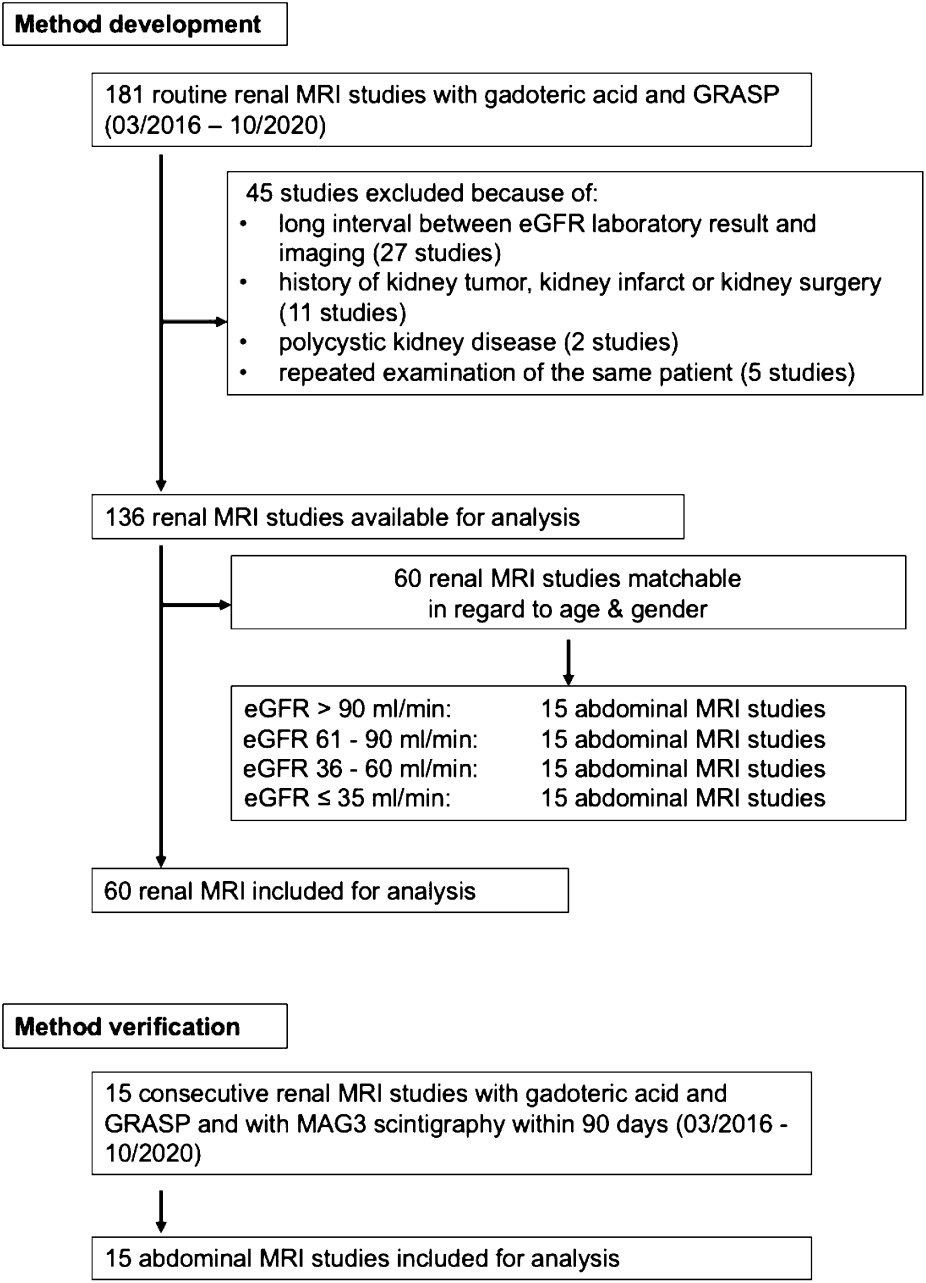
Study subject recruitment: method development and method verification

Subsequently, patients were grouped based on their kidney function defined by eGFR as calculated by the CKD-EPI formula^8^. The definition of the renal function groups closely followed the classification of chronic kidney disease as proposed by KDIGO (Kidney Disease: Improving Global Outcomes)^9^: ‘no impairment’, eGFR > 90 ml/min/1.73 m^2^; ‘mild impairment’, eGFR 60–89 ml/min/1.73 m^2^; ‘moderate impairment’, eGFR 36–59 ml/min/1.73 m^2^ and ‘severe impairment/renal failure’, eGFR 0–35 ml/min/1.73 m^2^, respectively. The final study population in the method development part consisted of 60 patients with 120 kidneys distributed into the four specified groups; all groups equal in patient number and matched for age and gender, Fig. 1.

For Method Verification, the local RIS was analogously searched for patients not included in the Method Development part of the study using identical time window for inclusion and identical inclusion and exclusion criteria. Additionally, a 99mTc-MAG3 dynamic renal scintigraphy within 90 days from the MRI examination was required. If an entry in the available clinical history of the patients suggested a possible change in renal function between the MRI examination and the scintigraphy (e.g. occurrence of ureterolithiasis), the patient was excluded. Differential renal function (DRF) was calculated by multiplying eGFR with the percentage of total renal function of the left and the right kidney, respectively, as measured by dynamic renal scintigraphy. The single kidney’s quantitative DRF (qDRF) defined whether this kidney was assumed to have no impairment of renal function (qDRF > 45 ml/min/1.73 m^2^), a mild/moderate impairment of renal function (qDRF 18–45 ml/min/1.73 m^2^) or a severe reduction in renal function/renal failure (qDRF <  18 ml/min/1.73 m^2^). The final study population in the method verification part consisted of 15 consecutive patients with 30 kidneys.

### MR parameters, acquisition and image reconstruction

MR examinations were performed with 1.5-T or 3.0-T scanners (Avanto, Skyra; Siemens Healthineers, Forchheim, Germany). 30 channel body phased-array coils were used together with 18 channel spine phased-array coils. A weight-adapted dose (0.2 ml per kg bodyweight) of gadoterate meglumine (Dotarem, Guerbet AG, Zurich, Switzerland) was injected intravenously using a power injector with an injection rate of 2 ml/s, followed by a bolus of 10 ml saline solution.

All DCE MRI studies were performed using a free-breathing approach. The dynamic contrast-enhanced acquisition used a fat-saturated T1-weighted radial stack-of-stars 3D GRE sequence with compressed-sensing and parallel-imaging reconstruction (GRASP) covering the entire kidneys with the following parameters: repetition time (3.29 ms), echo time (1.64 ms), field of view (320 mm × 320 mm), flip angle (12°), receiver bandwidth (600 Hz/Rx), in plane spatial resolution (1.1 mm × 1.1 mm), slice resolution (2.5 mm).

The GRASP sequence was acquired continuously in axial orientation for up to 350 s, consisting of a 20-s non-contrast imaging phase prior to contrast-material administration and a subsequent contrast-enhanced imaging phase of up to 330 s. GRASP performs the continuous acquisition with a radial stack-of-stars k-space sampling scheme in which radial spokes are stacked along the slice direction and rotated at a fixed angular increment given by the “golden angle”, increasing the angle of consecutive spokes by approximately 111.25°, which results in nearly uniform k-space coverage during the complete acquisition. This enables retrospective post-hoc reconstruction of image series with flexible temporal resolution by binning a certain number of consecutive spokes into individual time frames. GRASP uses an iterative compressed-sensing type of reconstruction that exploits temporal correlations between successive time frames to suppress undersampling artifacts and is thus able to reconstruct motion-resolved images from highly undersampled data 10. In addition to the inherent free-breathing imaging capability, retrospective respiratory gating may be enabled in the sequence. This functionality takes advantage of the self-navigation property of radial sampling and sorts the acquired data into the end-expiratory state 11, as this is the longest in duration. Furthermore, GRASP incorporates the receive-sensitivity profiles of the individual coil elements. A detailed technical description of the sequence and its imaging properties is provided in the corresponding technical studies [13].

In our study, a five-spoke reconstruction was used, resulting in a temporal resolution of 0.81–1.08 frames/sec for the first 90 s. The remainder of the GRASP acquisition was reconstructed with temporal resolutions of 0.14–0.17 frames/sec (6–7 s per frame).

### Renal dynamic scintigraphy

Renal dynamic scintigraphy examinations were performed employing SPECT/CT scanners (Symbia T6/T16 or Intevo T16, Siemens Healthineers, Erlangen, Germany). 99mTc-MAG3, a highly protein bound substance with primarily extraction by the proximal tubules, was used for estimation of tubular extraction rate. The administered activity was 90–110 MBq. Dynamic image acquisition in supine position was conducted for 30 min with start concomitant with tracer injection; 20 mg furosemide (Sanofi-Aventis SA, Vernier, Switzerland) was administered intravenously 12 min post injection. For image acquisition, a low energy high resolution collimator was used (matrix 64 × 64 pixel). Processing was performed using commercially-available postprocessing software application (syngo.via VB30, MI Application, Siemens Healthineers, Erlangen, Germany) resulting in split function parameters 1.5–2.5 min post injection in the parenchymal phase via ROI analysis. Estimation of the MAG3-TER was performed using the same software after measurement of count rates of 2 blood samples taken 20 and 25 min post injection.

### Quantitative assessment of GRASP DCE MR imaging

All GRASP datasets were analyzed to quantitatively evaluate the tracer’s passage through renal compartments. Under the assumption that a differentiated approach combining several independent measures would be more appropriate than a singular measurement and in order to avoid bias my renal hypoperfusion, a threefold approach was chosen with evaluation of (a) glomeruli perfusion, (b) tubular concentration, and (c) collecting system excretion. All post-processing was performed on a commercially-available software application (syngo.via VB30; Siemens Healthineers, Erlangen, Germany). Regions-of-interest (ROI) were drawn on renal cortex, medullary pyramids, and pelvises, each on three craniocaudal levels (upper pole, midsection, lower pole) covering an area of at least 0.5 cm^2^ as well as within abdominal aorta covering an area of at least 1 cm^2^ at the level of the renal artery origin. Each ROI was manually drawn on a single frame and automatically multiplicated for all other frames. All ROI placements were individually performed for right kidney anatomy as well as left kidney anatomy. ROIs were drawn by a board-certified radiologist with 2 years of experience in abdominal imaging (X.X.). ROI placements were reviewed by an expert in abdominal imaging with more than 20 years of experience in abdominal imaging (X.X.X.) If patient motion occurred, every ROI on every frame was checked to ensure correct placement. Multilevel ROIs were averaged. Normalized time-intensity parameters were extracted.

#### Glomeruli perfusion:

**Fig. 2:**
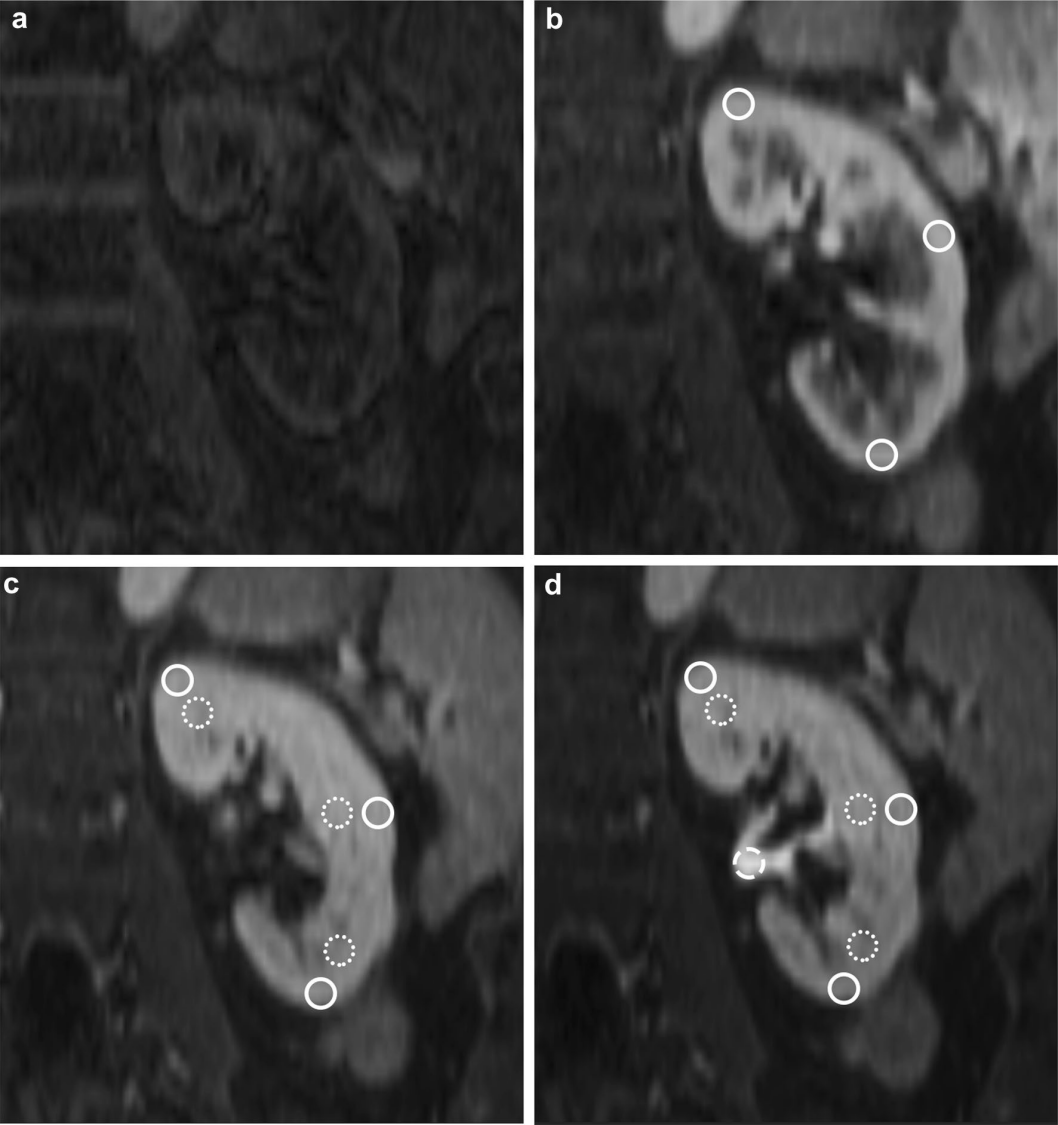
Visualization of contrast bolus passage through various renal compartments in a 65-year-old female patients with mild renal function impairment: **a** non-contrast GRASP image (Time = 0 s); **b** glomeruli perfusion with contrast peak in the renal cortex (solid ROI, Time = 35 s); **c** tubuli concentration with equalization of contrastation in the renal cortex and medullary pyramids (solid and dotted ROIs, respectively, Time = 75 s); **d** collecting system exclusion with contrast rise in the collecting system (dashed ROI, Time = 140 s)

The majority of renal blood flow is directed to the glomeruli, of which about 90% are located within the cortex^13^. The ROIs within the renal cortex can thus be characterized as surrogate for a mainly microvascular compartment. Within the glomeruli, the primary urine is formed as product of ultrafiltration of blood. Since gadoterate meglumine is freely filtrated^4^, its concentration in the primary urine can be assumed to be closely similar to its concentration in blood plasma, Fig. 2.

#### Tubular concentration:

The primary urine passes from the glomeruli to the tubules, the majority of which are located in the medullary pyramids. The ROIs within the medullary pyramids can therefore be characterized as surrogate for a mostly tubular compartment. Here, water is being extracted, leading to a rise in the gadoterate meglumine concentration. Since renal medullary pyramids are less vascularized compared to renal cortex, an increase of signal intensity of the medullary pyramids above the level of the cortex indicates the accumulation of filtrated and at least partially concentrated gadoterate meglumine in the tubules, Fig. 2. The increasing concentration of gadoterate meglumine expressed as the slope of the intensity curve of the medullary pyramids can be perceived as velocity of gadoterate meglumine filtration with simultaneously occurring water reabsorption. Slope quantification (Slope_Tubuli_) was determined on normalized time-intensity curves within a time window which encompassed 10 s before and 10 s after the timepoint of crossing between the medullary and the normalized cortical intensity curves (Time_Cortex=Pyramid_), Fig. 2.

#### Collecting system excretion:

Lastly, the concentrated urine is collected in the renal pelvises. The ROIs in the renal pelvis can therefore be defined as surrogate for excretion of concentrated gadoterate meglumine. Rise of signal intensity in the renal pelvis indicated excretion of contrast agent (Time_Collecting System_), Fig. 2.

### Statistical analysis

All quantitative results evaluating timepoint of crossing of cortical intensity and medullary pyramid intensity curves (Time_Cortex=Pyramid_) in sec, slope of the medullary curve (Slope_Tubuli_) in sec^−1^, and timepoint of signal intensity rise in the renal pelvises (Time_Collecting System_) in sec, are presented as mean ± standard deviation (SD). Time points are referenced against the arrival of contrast agent in the renal arteries.

For method development, primarily ANalysis Of VAriance (ANOVA) methodology was used for statistical comparison. The estimated glomerular filtration rate (eGFR) (ml/min/1.73 m^2^) was defined as an independent factor for each analysis; Bonferroni post-hoc corrections were applied for fixed factor “renal function group”; values for Time_Cortex=Pyramid_ (sec)_,_ Slope_Tubuli_ (sec^−1^)_,_ and Time_Collecting System_ (sec) were successively selected as the dependent variable. The statistical investigation employed SPSS (SPSS Statistics 21, SPSS Inc., Chicago, IL) and a *p*-value <  0.05 was considered statistically significant.

Secondarily, receiver operating characteristics (ROC) analyses with consecutive Youden tests were conducted and yielded cut-off values for Time_Cortex=Pyramid_ (sec)_,_ Slope_Tubuli_ (sec^−1^)_,_ and Time_Collecting System_ (sec) to best discriminate between the following renal function groups: “no impairment” vs. “mild & moderate impairment” as well as “no & mild & moderate impairment” vs. “severe reduction in renal function/renal failure”.

For method verification, each kidney was assigned to the appropriate renal function group using the cut-off values determined during method development for Time_Cortex=Pyramid_ (sec)_,_ Slope_Tubuli_ (sec^−1^)_,_ and Time_Collecting System_ (sec); the two groups “mild impairment”/“moderate impairment” were combined for method verification assessment. In case of discordant group assignment based on the three cutoff values, the group indicated by two of the three measures was selected (majority principle). The reference standard for comparison was determined by qDRF as defined by means of dynamic renal scintigraphy. The method’s positive predictive value (PPV), negative predictive value (NPV) and accuracy were calculated focusing on detection of renal function (RF) group: ‘no impairment’, ‘mild/moderate reduction of RF’ and ‘severe reduction of RF/renal failure’.

## Results

### Demographics

The participants assigned to the method development population distributed into the four renal function groups were matched in regard to mean age, with a slight male predominance within each group (Table 1). The consecutively accrued participants in the method verification population distributed into the three “renal function groups” showed a slight female predominance within each group (Table 2).

**Table 1:** Demographics of the method development population

Renal function group (eGFR range)	Number of patients (female: male patients)	eGFR (mean ± std. dev., range, in ml/min/1.73 m^2^)	Age (mean ± std. dev., range, in years)
‘No impairment’ (>90 ml/min/1.73 m^2^)	15 (5:10)	98.5 ± 7.7 (91–123)	64.1 ± 11.2 (28–76)
‘Mild impairment’ (61–90 ml/min/1.73 m^2^)	15 (5:10)	75.1 ± 8.9 (61–89)	69.1 ± 11.3 (38–83)
‘Moderate impairment’ (35–60 ml/min/1.73 m^2^)	15 (5:10)	46.6 ± 6.6 (36–58)	66.3 ± 10.0 (46–81)
‘Severe impairment’ (<35 ml/min/1.73 m^2^)	15 (5:10)	24.2 ± 9.4 (5–33)	67.7 ± 13.9 (29–85)

**Table 2:** Demographics of the method verification population

Renal function group (eGFR range)	Number of kidneys (female: male patients)	qDRF (mean ± std. dev., range, in ml/min/1.73 m^2^)	Age (mean ± std. dev., range, in years)
‘No impairment’ (>90 ml/min/1.73 m^2^)	9 (5:4)	62.7 ± 10.6 (46.6–83.3)	47.1 ± 17.1 (23–75)
‘Mild/moderate impairment’ (35–90 ml/min/1.73 m^2^)	12 (9:3)	29.1 ± 4.4 (22.4–35.7)	57.4 ± 17.9 (22–75)
‘Severe impairment’ (<35 ml/min/1.73 m^2^)	9 (6:3)	13.6 ± 3.2 (8.2–17.7)	66.6 ± 20.2 (22–84)

### Method development

**Fig. 3:**
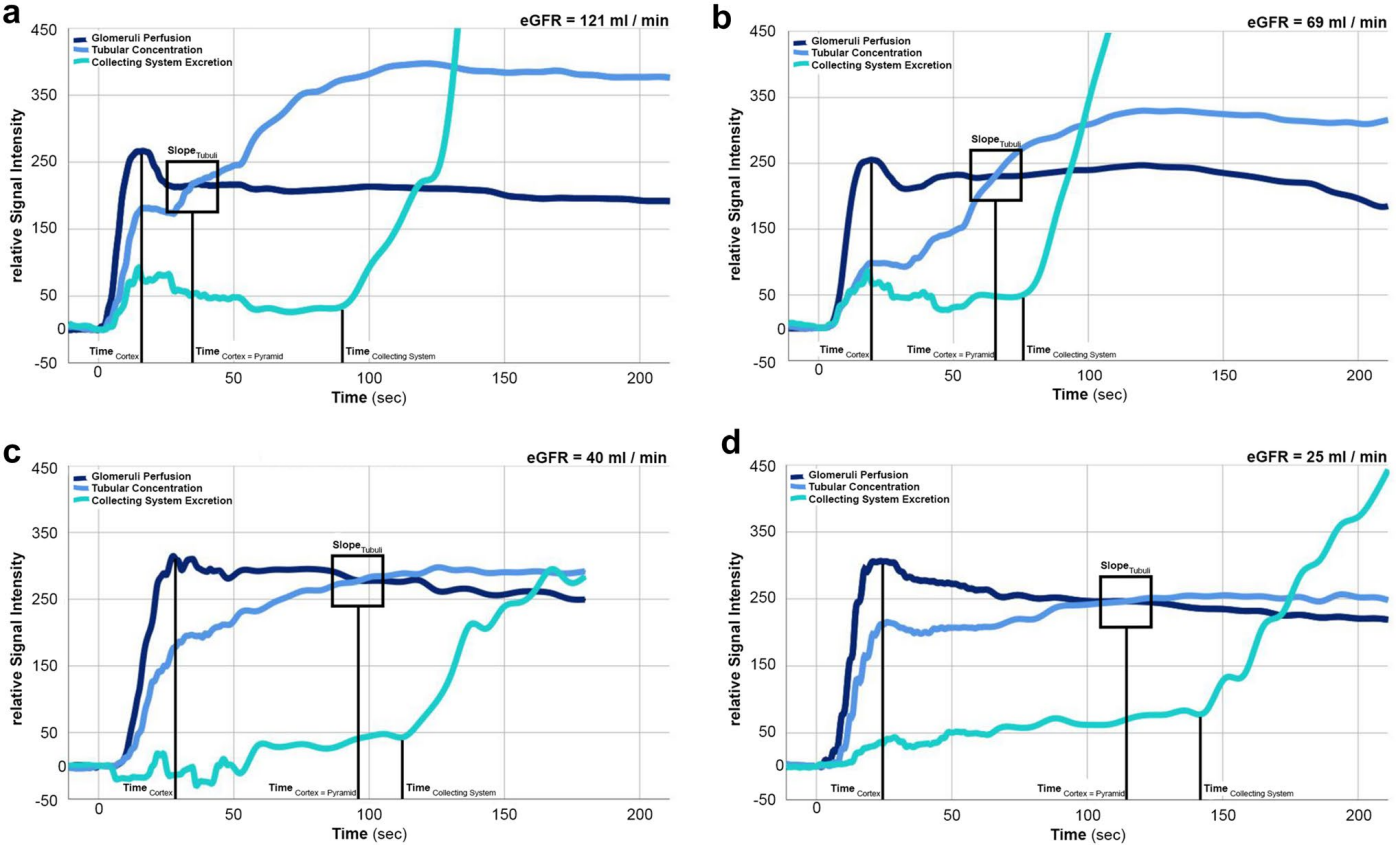
Visualization of contrast bolus passage through various renal compartments in four different 65-year-old male patients with deteriorating renal function. Note the increasingly delayed crossing of cortical and medullary intensity curves (Time_Cortex=Pyramid_), flattening of the medullary curve (Slope_Tubuli_) and delayed increase in measured excretion (Time_Collecting System_) into the real collecting system as the renal function deteriorates

The quantification of GBCA passage through vascular, tubular and collecting system compartments showed a general tendency of increasingly delayed crossing of cortical and medullary intensity curves, flattening of the medullary curve and delayed increase in excretion into the real collecting system as renal function deteriorated. These trends exhibited partial statistical significance as shown in Table 3, Fig. 3.

**Table 3:** Quantification of contrast material passage through vascular, tubular and collecting system compartments

Renal function group (eGFR range)	Time_Cortex=Pyramid_ (mean ± std. dev., in sec)	Slope_Tubuli_ (mean ± std. dev., in sec^−1^)	Time_Collecting System_ (mean ± std. dev., in sec)
‘No impairment’ (>90 ml/min/1.73 m^2^)	71.4 ± 20.8	2.6 ± 1.5	90.0 ± 33.8* (*p* < 0.001)
‘Mild impairment’ (61–90 ml/min/1.73 m^2^)	74.7 ± 22.8	2.1 ± 1.5* (*p* < 0.001)	110.9 ± 22.2* (*p* < 0.001)
‘Moderate impairment’ (35–60 ml/min/1.73 m^2^)	93.1 ± 33.2	1.3 ± 0.8	128.9 ± 28.6
‘Severe impairment’ (<35 ml/min/1.73 m^2^)	121.8 ± 43.7* (*p* < 0.001)	0.5 ± 0.6* (*p* < 0.001)	139.4 ± 20.2* (*p* < 0.001)

Asterisk indicates statistically significant differences of the indicated group in contrast to all three other groups, significance level *p* <  0.05

Receiver operating characteristics (ROC) analyses with consecutive Youden tests calculated cutoff values for Time_Cortex=Pyramid_, Slope_Tubuli_, and Time_Collecting System_ to best differentiate ‘no impairment’ from ‘mild/moderate impairment’ as well as ‘no/mild/moderate impairment’ from ‘severe impairment with an AUROC of 0.83 and 0.81, respectively, Table 4.

**Table 4:** ROC analyses with consecutive Youden-calculated cutoff values for each of the three parameters quantifying contrast material passage through vascular, tubular and collecting system compartments

Renal function group (eGFR range)	Time_Cortex=Pyramid_ (in sec)	Slope_Tubuli_ (in sec^−1^)	Time_Collecting System_ (in sec)	ROC_AUC_ (sensitivity/specificity)
‘No Impairment’ (>90 ml/min/1.73 m^2^) vs ‘Mild/moderate impairment’ (35–90 ml/min/1.73 m^2^)	>84.8	<2.0	>97.9	0.83 (0.76/0.86)
‘No/mild/moderate impairment’ (>35 ml/min/1.73 m^2^) vs ‘Severe Impairment’ (<35 ml/min/1.73 m^2^)	>99.7	<0.9	>128.5	0.81 (0.91/0.69)

### Method verification

**Fig. 4:**
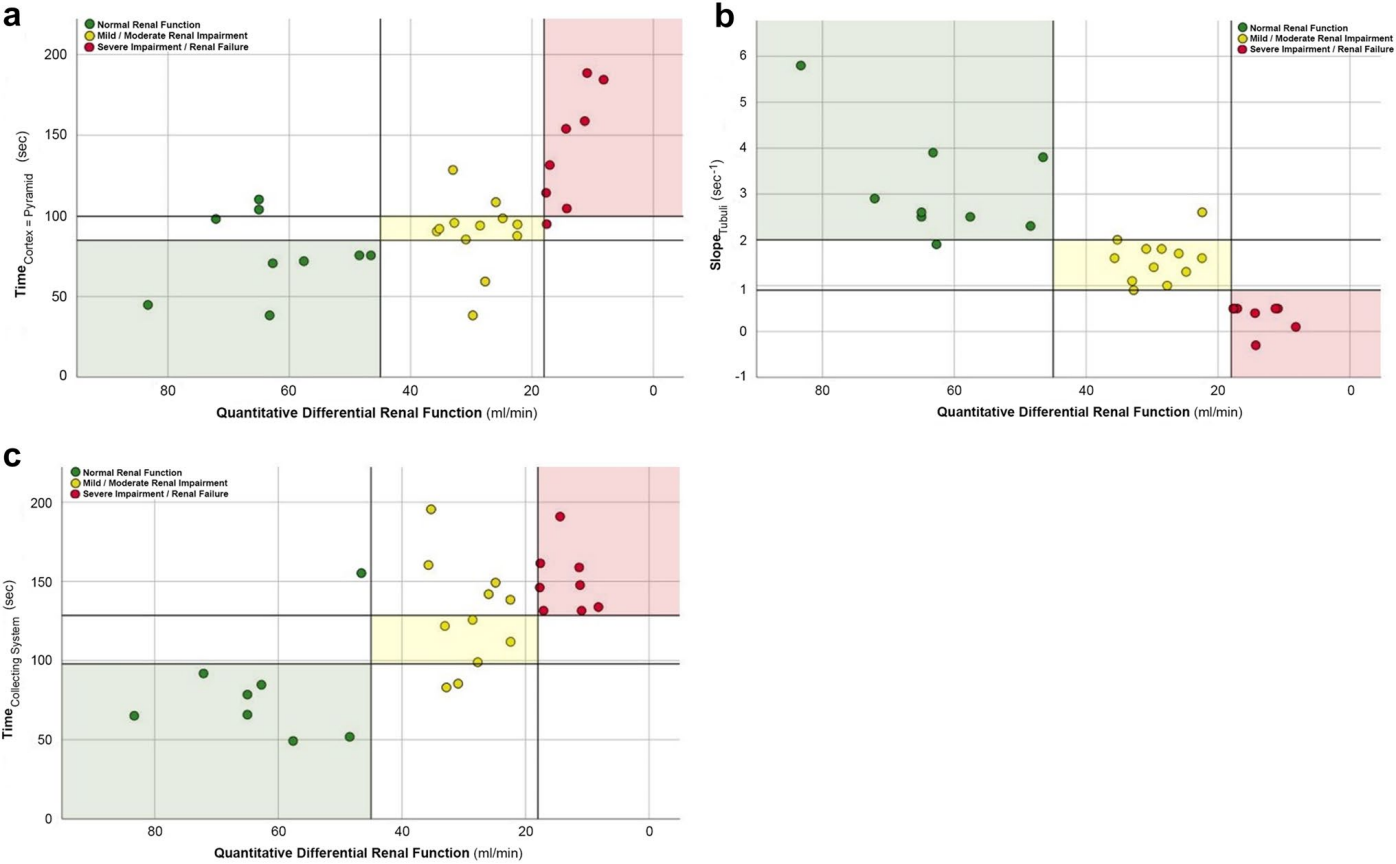
Method Verification: each kidney was assigned to the renal function group defined by the cut-off values for **a** Time_Cortex=Pyramid_ (sec), **b** Slope_Tubuli_ (sec^−1^)_,_ and **c** Time_Collecting System_ (sec) is seen on the y-axes; the two groups “mild impairment”/“moderate impairment” were combined for method verification. The x-axes show the quantitative differential renal function (ml/min) as determined by renal dynamic scintigraphy; markers of colorized kidneys placed in identically colorized shaded areas have been correctly characterized in regard underlying renal function

Kidneys were assigned to the appropriate renal function group using the three cut-off values determined during method development for Time_Cortex=Pyramid_ (sec), Slope_Tubuli_ (sec^−1^), and Time_Collecting System_ (sec) and were referenced against dynamic renal scintigraphy. 29/30 kidneys (96.7%) were assigned to the correct renal function group, resulting in test characteristics shown in Table 5, Fig. 4.

**Table 5:** 29/30 kidneys (96.7%) were assigned to the correct group “renal function group”; resulting in test characteristics

Renal function group (eGFR range)	PPV	NPV	Accuracy
‘No Impairment’ (>90 ml/min/1.73 m^2^)	1.00	1.00	1.00
‘Mild/Moderate impairment’ (35–90 ml/min/1.73 m^2^)	1.00	0.95	0.97
‘Severe impairment’ (<35 ml/min/1.73 m^2^)	0.90	1.00	0.97

## Discussion and conclusions

This study sought to assess whether the extraction of split RF is a feasible byproduct of routine abdominal MRI examinations when compressed sensing DCE-MRI with GRASP is applied. Our results indicate that individual kidneys can be characterized to having normal RF, mild/moderate impairment of RF or severe impairment of RF/renal failure by the method developed and verified in this study. While each of these categories included a certain range of RF parameters and the method developed in the study did not yield an exact qDRF as known to be produced by SPECT, the results, nonetheless, may be helpful in clinical decision-making:

 − In patients considered for radical tumor nephrectomy, the American Urological Association’s Renal Cancer Guidelines (2021) demand the expected postsurgical baseline eGFR to be at least 45 ml/min/1.73 m^2^, otherwise partial nephrectomy should be considered^14^.
 − In a patient prior to living kidney donation, any evidence of reduction of RF of one of both kidneys might result in the need of further preoperative investigation, and impacts which kidney may be selected for explanation beyond anatomical considerations such as vascular and morphologic features of the collecting systems as well as presence of pre-existing focal renal lesions^15^.

While an emphasis of this study was to show that RF information may be feasibly extracted as byproduct of routine abdominal MRI examinations, neither the concentration of administered GBCA nor the choice of imaging sequence were optimized for linear MR signal-GBCA concentration relationships and subsequent kinetic modeling as reported previously to be an essential prerequisite for quantitative analyses^5^. Therefore, a different approach was developed which does not require estimation of GBCA concentration. Instead, extraction of time intensity curves and their respective quantitative parameters was performed, thereby measuring passage of GBCA through the renal cortex, the medullary pyramids and the renal pelvis collecting system as surrogates for a mainly vascular compartment, a mainly tubular compartment and collecting basin compartments, respectively. No quantitative estimation of GFR was obtained with this method, however, differentiation of several degrees of renal function impairment was achieved. Precise measurements of time points and upslope steepnesses were enabled by using the high temporal and spatial resolution offered by GRASP.

Patients with severely impaired renal function or renal failure showed a substantially temporal delay to reach cortical and medullary enhancement compared to patients with normal renal function. This observation likely correlates with presence and amount of renal glomeruli fibrosis and subsequent hypoperfusion, both known aspects of the pathophysiology of chronic kidney disease and present not only in primary hypoperfusion-associated kidney injury but also in diabetic or obstructive chronic kidney disease^16^. However, this temporal delay reached significance only when comparing the normal renal function group to the population with known renal failure. Therefore, additional measures such as the slope of GBCA concentration increase in the tubuli and the temporal delay until GBCA was detected in the collecting system were included in this multifactorial evaluation. By including all three compartments into the GRASP-based analyses, it is reasonable to assume that a global kidney function assessment was performed beyond solely focusing on renal perfusion.

This study has limitations which have to be addressed:

Primarily, a retrospective study with limited size of study population limits its generalization. Therefore, larger studies are needed to further validate these results. However, by selecting this approach, the evaluated patients represent a cross-section of renal impairment frequently encountered in abdominal MR imaging. As a feasibility study emphasizing the ability to extract renal function information as a byproduct of routine MR-imaging, this study design showed to be sufficient.

Secondarily, the use of ROIs instead of whole organ or whole compartment measurements, as can be achieved by whole-organ segmentation represents a limitation. Renal size and cortical thickness did therefore not contribute to the assessment of renal function.

Further, dynamic renal scintigraphy was carried out using an injection of the tubular extraction agent MAG3 with estimation of MAG3-TER resulting in kidney function estimates reflecting tubular extraction. Furthermore, dynamic renal scintigraphy included injection of furosemide 12 min after tracer injection, while protocols for abdominal MRI did not include furosemide application. For this reason, direct comparison of tracer kinetic curves and signal intensity curves was omitted. However, it is reasonable to assume that a comparison between both modalities by means of several degrees of renal function impairment is appropriate.

The use of GBCA has been linked to nephrogenic systemic fibrosis (NSF). However, it should be noted that the above mentioned clinical scenarios of nephrectomy include patients who typically have known eGFR > 30 ml/min/1.73 m^2^ and that application of macrocyclic GBCA should not be withheld in this patient group, according to latest ACR recommendations [18].

In summary, this pilot study showed that high temporal and spatial resolution GRASP MR imaging allows to identify several degrees of renal function impairment using routine clinical imaging with a high degree of accuracy which may be helpful in clinical decision-making, in particular for patients undergoing unilateral nephrectomy. Furthermore, it will be helpful to promote the application of DCE MRI in evaluating renal function.

## Key findings

- Several levels of renal function impairment can successfully be determined on routine abdominal MRI imaging when using GRASP, correlated with dynamic renal scintigraphy.
- Post-hoc increase in temporal and spatial resolution of GRASP allows precise quantification of passage of gadolinium-based contrast material as tracer through vascular, tubular and collecting system compartments in sub-second increments.

## Importance

Assessing renal function impairment during routine clinical imaging with a high degree of accuracy may be helpful in clinical decision-making, in particular for patients undergoing unilateral nephrectomy.

## Abbreviations

GBCA: Gadolinium-based contrast agent
GRASP: Golden-angle radial sparse parallel
GFR: Estimated glomerular filtration rate
SPECT: Single photon emission-computed tomography
MAG3: Mercaptoacetyltriglycine
TER: Tubular extraction rate
ROI: Region of interest
RSI: Radiology information system
CKD-EPI: Chronic Kidney Disease Epidemiology Collaboration
KDIGO: Kidney Disease: Improving Global Outcomes
qDRF: Quantitative Differential renal function
ROC: Receiver operating characteristics
RF: Renal function
